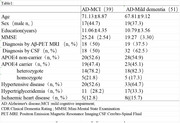# Protocol for a multicenter, prospective, real‐world study: Lecanemab in patients with early Alzheimer's disease(LEAD) in China

**DOI:** 10.1002/alz70859_101524

**Published:** 2025-12-25

**Authors:** Hui Zhao, Limoran Tang, Dan Yang, Nihong Chen, Yun Xu

**Affiliations:** ^1^ Department of Neurology, Affiliated Drum Tower Hospital, Nanjing University Medical School, Nanjing China; ^2^ Department of Neurology, Afflicated Drum Tower Hospita, NANJING, JIANGSU China; ^3^ Department of Neurology, Nanjing Drum Tower Hospital, NANJING, JIANGSU China; ^4^ Department of Neurology, Nanjing First Hospital, Nanjing Medical University, NANJING, JIANGSU China; ^5^ Nanjing Drum Tower Hospital Clinical College of Nanjing Medical University, Nanjing, Jiangsu China

## Abstract

**Background:**

Lecanemab, a humanized IgG1 monoclonal antibody that targets soluble aggregated Aβ species (protofibrils), has demonstrated robust brain fibrillar amyloid reduction and slowing of clinical decline in early Alzheimer's disease(AD) and received its approval for this indication in the USA 2023 and China 2024. However, reports published of real‐world data on this therapy are still very limited, espcially in China. LEAD is an investigator‐ led, multicenter, prospective, real‐world study in 20 hospitals in Jiangsu Province, China. The primary objective was to assess the effectiveness of lecanemab under real‐world clinical practice in early Alzheimer's diseasepatients in China. The secondary objectives included evaluating the safety of lecanemab in Chinese patients.

**Method:**

This prospective cohort trial included early AD (mild cognitive impairment or mild dementia due to AD) patients having an indication for lecanemab. Each participant had evidence of amyloid on positronemission tomography (PET) or by cerebrospinal fluid testing. Baseline data on demographics, medical history, cognitive score, plasma biomarkers, Apoe and MR indicators were assessed and a treatment with intravenous lecanemab (10 mg per kilogram of body weight each 2 weeks) was started. The patients will be followed up, attend safety visits, provide blood samples to measure AD biomarkers at months 3, 6 and 12. AD blood biomarkers was detected using chemiluminescence. All cognitive assessments will be repeated at month 6 and 12, structural MR will be noted at month 1,1.5,3.5 and 6. The last study visit will be at month 12, when all baseline measurements will be repeated. The study was approved by the Drum Tower Hospital Research Ethics Committee (2024‐746‐01).

**Result:**

This study is onging, 90 subjects have been recruited. Currently in the data extraction phase. Baseline data collection of the subjects is shown in Table 1. All patients will complete 6 months of treatment in July 2025. Updated result will be available for the conference.

**Conclusion:**

The LEAD project is a multi‐center, real‐world study that will determine the therapeutic efficacy of lecanemab in alleviating clinical symptoms and pathological aspects in Alzheimer's disease patients in Jiangsu, China, as well as monitor adverse effects, in order to assist the treatment of more Asian AD patients.